# Covid-19 pandemics effects on postpartum depression in the Hungarian Baby-Mother-Father Unit

**DOI:** 10.1192/j.eurpsy.2024.491

**Published:** 2024-08-27

**Authors:** G. M. Mező, C. Budinszki, T. Kurimay

**Affiliations:** Family Centred Buda Mental Health Centre, Saint John’s Central Hospital, Budapest, Hungary

## Abstract

**Introduction:**

Our Baby-Mother-Father Unit program in Saint John’s Central Hospital (Budapest) offers mothers and fathers a unique opportunity to get better, receive psychiatric care (hospitalization or outpatient) without being separated from their babies. The Covid-19 pandemic had a strong impact on the whole population, including the parents of babies. During everyday operation the whole team experienced the increased need for health care, but we were not aware of the exact number of this change.

Postpartum psychiatric conditions have two main categories that stand out: postpartum psychosis and postpartum depression. As there are better quantifiable tools for measuring depression and strong scientific evidence supporting that the pandemic having increased mood disorders’ intensity and numbers (Chen et al., 2022; Harrison et al., 2023), postpartum depression was chosen as the locus of investigation. Due to the respectively high numbers of parents with babies showing up at our Unit, we wished to get a clearer picture on pandemics effects on these people.

**Objectives:**

Getting a more clear picture of pandemics effects on our Baby-Mother-Father Unit care. Defining numbers of patients, interactions and comparing test results of depression scales before and after the pandemic.

**Methods:**

A retrospective study of years 2019 and 2022 was performed. The total number of patients (2019: 173, 2022: 278) and the total number of documented patient-doctor/psychologist interactions (2019: 963, 2022: 1919) were measured. Depression scales’ (BDI, EPDS, PHQ-9), hopelessness scales (HS) results were compared. Due to our samples not showing normal distribution, a deeper analysis of test result categories was carried out by using Mann-Whitney test.

**Results:**

The results showed that depression (BDI: W=3165,5 p=0,17; EPDS: W=1693, p=0,42; PHQ-9: W=2502, p=0,39) and hopelessness (RS: W=976,5, p=0,52) average points seem quite constant regardless of the pandemic and showed no significant differences. More detailed data analysis of result categories revealed pattern-like differences, which might tell us more about the subjective experiences of the individuals. The number of patients and patient-doctor/psychologists interactions increased dramatically. Furthermore the number of individual therapeutic sessions rose greatly (2019: 359; 2022: 1182), along with parents receiving therapeutic care (2019: 40, 2022: 95).

**Conclusions:**

From our findings, assumptions can be made that besides the obvious rise of numbers of patients and interactions, during the pandemic postpartum depression’s and hopelessness’ structure changed.

**Disclosure of Interest:**

None Declared

